# Phylogeny and Taxonomy of *Archaea*: A Comparison of the Whole-Genome-Based CVTree Approach with 16S rRNA Sequence Analysis

**DOI:** 10.3390/life5010949

**Published:** 2015-03-17

**Authors:** Guanghong Zuo, Zhao Xu, Bailin Hao

**Affiliations:** 1 T-Life Research Center and Department of Physics, Fudan University, 220 Handan Road, Shanghai 200433, China; E-Mail: ghongzuo@gmail.com; 2 Thermo Fisher Scientific, 200 Oyster Point Blvd, South San Francisco, CA 94080, USA; E-Mail: xuzh.fdu@gmail.com

**Keywords:** Archaea, phylogeny and taxonomy, 16S rRNA analysis, whole-genome comparison, alignment free, CVTree

## Abstract

A tripartite comparison of *Archaea* phylogeny and taxonomy at and above the rank order is reported: (1) the whole-genome-based and alignment-free CVTree using 179 genomes; (2) the 16S rRNA analysis exemplified by the All-Species Living Tree with 366 archaeal sequences; and (3) the Second Edition of *Bergey’s Manual of Systematic Bacteriology* complemented by some current literature. A high degree of agreement is reached at these ranks. From the newly proposed archaeal phyla, *Korarchaeota*, *Thaumarchaeota*, *Nanoarchaeota* and *Aigarchaeota*, to the recent suggestion to divide the class *Halobacteria* into three orders, all gain substantial support from CVTree. In addition, the CVTree helped to determine the taxonomic position of some newly sequenced genomes without proper lineage information. A few discrepancies between the CVTree and the 16S rRNA approaches call for further investigation.

## 1. Introduction

Prokaryotes are the most abundant and diverse creatures on Earth. The recognition of *Archaea* as one of the three main domains of life [1,2] was a milestone in the development of biology and a great success of using the 16S rRNA sequences as molecular clocks for prokaryotes, as suggested by Carl Woese and coworkers [3,4]. The Second Edition of *Bergey’s Manual of Systematic Bacteriology* [5] (hereafter, the Manual), a magnificent work of more than 8000 pages, took 12 years (2001–2012) to complete and is being considered by many microbiologists as the best approximation to an official classification of prokaryotes [6]. As stated in the Preface to vol. 1 of the Manual, these volumes “follow a phylogenetic framework based on analysis of the nucleotide sequence of the small ribosomal subunit RNA, rather than a phenotypic structure.” However, the “congruence” of phylogeny and taxonomy on the basis of 16S rRNA sequence analysis raises a question of principle, namely the necessity of cross-verification of whether the present classification is capable of providing a natural and objective demarcation of microbial organisms.

The answer comes with the advent of the genomic era. A whole-genome-based, alignment-free, composition vector approach to prokaryotic phylogeny, called CVTree [7,8,9,10,11,12], has produced robust phylogenetic trees that agree with prokaryotic taxonomy almost at all taxonomic ranks, from domain down to genera and species, and more importantly, many apparent disagreements have disappeared, with new taxonomic revisions appearing. In fact, all published taxonomic revisions for prokaryotes with sequenced genomes have added to the agreement of CVTree with taxonomy. A recent example from the domain *Archaea* was the reclassification of *Thermoproteus neutrophilus* to *Pyrobaculum neutrophilum* [13].

In this paper, we study *Archaea* phylogeny across many phyla. This is distinct from the phylogeny of species in a narrow range of taxa, e.g., that of vertebrates (a subphylum) or human *versus* close relatives (a few genera). Accordingly, the phylogeny should be compared with taxonomy at large or, as Cavalier-Smith [14] put it, with “mega-classification” of prokaryotes, focusing on taxonomy of higher ranks. Although in taxonomy, the description of a newly discovered organism necessarily starts from the lower ranks, higher rank assignments are often incomplete or lacking. At present, the ranks above class are not covered by the Bacteriological Code [15,16]. The number of plausible microbial phyla may reach hundreds, and archaeal ones are among the least studied. According to the 16S rRNA analysis, the major archaeal classes and their subordinate orders have been more or less delineated. Therefore, in order to carry out the aforementioned cross-verification, we make an emphasis on higher ranks, such as phyla, classes and orders. A study using 179 Archaea genomes provides a framework for the further study of lower ranks.

## 2. Material and Method

Publicly available Archaea genome sequences are the material for this study. At present, more than 30,000 prokaryotic genomes have been sequenced [17], among which, about 16,000 have been annotated [18]. These numbers keep growing and make whole-genome approaches more than ever feasible.

As of the end of 2014, there were 165 *Archaea* genomes released on the NCBI FTP site [19]. These genomes with corresponding lineage information from NCBI taxonomy were part of the built-in database of the CVTree web servers [20,21]. A search of NCBI databases revealed 14 more archaeal genomes; these were uploaded to the web server at run time. Archaea genomes listed in the EBI Genome Pages [22] were all included. A full list of these 179 genomes with accession numbers is given in the Appendix.

A whole-genome-based phylogeny avoids the selection of sequence segments or orthologous genes. It must be alignment-free, due to the extreme diversity of prokaryotic genome size and gene content. Our way of implementing alignment-free comparison consists of using *K*-peptide counts in all protein products encoded in a genome to form a raw “composition vector” (CV). The raw CV components then undergo a subtraction procedure in order to diminish the background caused by neutral mutations, hence to highlight the shaping role of natural selection [23]. Using whole genomes as input data also helps to circumvent the problem of lateral gene transfer (LGT), as the latter is merely a mechanism of genome evolution together with lineage-dependent gene loss. Being a nightmare for single- or few-protein-based phylogeny, LGT may even play a positive role in whole-genome approaches, as it takes place basically in shared ecological niches [24] and among closely-related species [25]. Plasmid genomes were excluded from our input data, thus further reducing plasmid-mediated LGT. Using whole genome input and the alignment-free method also makes CVTree a parameter-free approach. In other words, given the genomes, phylogenetic trees are generated without any adjustment of the parameters or the selection of sequence segments.

As the CVTree methodology has been elucidated in many previous publications (see, e.g., [7,8,9,10,11,12]) and a web server was released twice in 2004 [26] and 2009 [20], we will not discuss the methodological aspects of CVTree here. However, it should be understood that the peptide length *K*, though looking like a parameter, does not function as a parameter. For a discussion on the role of *K* and why *K* = 5*,* 6 leads to the best results, we refer to a recent paper [27]. All CVTree figures shown in this paper were generated at *K* = 6. In this paper, the term CVTree is used to denote the method [7,8,9,10,11,12,27], the web server [20,21,26] and the resulting tree; see, e.g., [28].

Traditionally, a newly generated phylogenetic tree is subject to statistical re-sampling tests, such as bootstrap and jackknife. CVTree does not use sequence alignment. Consequently, there is no way to recognize informative or non-informative sites. Instead, we take all of the protein products encoded in a genome as a sampling pool for carrying out bootstrap or jackknife tests [7]. Although it was very time-consuming, CVTrees did pass these tests well [11]. However, successfully passing of statistical re-sampling tests only informs about the stability and self-consistency of the tree with respect to small variations of the input data. It is by far not a proof of the objective correctness of the tree. Direct comparison of all branchings in a tree with an independent taxonomy at all ranks would provide such a proof. The 16S rRNA phylogeny cannot be verified by Bergey’s taxonomy, as the latter follows the former. However, the agreement of branchings in CVTree with Bergey’s taxonomy would provide much stronger support to the tree, as compared to statistical tests. This is the strategy we adopt for the CVTree approach.

There are two aspects of a phylogenetic tree: the branching order (topology) and the branch lengths. Branching order is related to classification and branch length to evolution time. Calibration of branch lengths is always associated with the assumption that the mutation rate remains more or less a constant across all species represented in a tree, an assumption that cannot hold true in a large-scale phylogenetic study, like the present one. Therefore, branching order in trees is of primary concern, whereas calibration of branch lengths makes less sense. Accordingly, all figures in this paper only show the branching scheme without the indication of branch lengths and bootstrap values.

Branching order in a tree by itself does not bring about taxonomic ranks, e.g., class or order. The latter can be assigned only after comparison with a reference taxonomy, which is not a rigid framework, but a modifiable system. Though there is a dissimilarity measure in the CVTree algorithm, it is not realistic to delineate taxa by using this measure, at least for the time being. Even if defined in the future, it must be lineage dependent. For example, it cannot be expected that the same degree of dissimilarity may be used to delineate classes in all phyla. In addition, monophyly is a guiding principle in comparing branching order with taxonomy. Here, monophyly must be understood in a pragmatic way, restricted to the given set of input data and the reference taxonomy. If all genomes from a taxon appear exclusively in a tree branch, the branch is said to be monophyletic.

In order to effectively deal with several thousands of genomes in a run, we have parallelized the CVTree algorithm and moved the web server to a computer cluster with 64 cores. The new CVTree3 web server [21] is capable of producing trees with several thousands of leaves in a few minutes for a range of *K*-values, say for *K* = 3 to 7. In addition, the CVTree3 web server has the following advanced features:

(1) CVTree3 is equipped with an interactive tree display, which allows collapsing or expanding the tree branches at the disposal of the user. The user may concentrate on an interested taxon by submitting an enquiry; only the neighborhood of the taxon is expanded and all of the rest collapsed properly, keeping the topology unchanged. Here, “collapsing” means replacing a whole branch by a single leaf. Usually, a collapsed branch is labeled by the name of the highest common taxon followed by the number of strains it represents. For example, *<*C*>*Methanococci{12} denotes a class-level monophyletic branch containing 12 leaves. If a taxon name is seen in two (or more) collapsed branches, such as *<*C*>*Classname{3/12} and *<*C*>*Classname{9/12}, then the taxonomically monophyletic class does not correspond to a single branch in the collapsed tree.
(2) The web server reports “convergence statistics” of all tree branches, *i.e*., a list of all monophyletic and non-monophyletic taxa at all taxonomic ranks for every *K*-value. For example, the first two lines of the report read:
  <*D*>*Archaea*{165}*− −K*5*K*6*K*7*−*
  <*D*>*Bacteria*{2707}* − −K*5*K*6 *− −*
  (Numerals in curly brackets tell the number of organisms present in a collapsed branch.) Therefore, the two domains *Archaea* and *Bacteria* are both well defined as monophyletic branches at *K* = 5 and 6. We note that in the statistics, only genomes with complete lineage information are counted. The example project referred to in this paper contained, in addition, 14 archaeal and 143 bacterial genomes with one or more “unclassified” rank in the lineage. Therefore, in total {165 + 14}= 179 Archaea and {2707+243}= 2850 Bacteria genomes were used. The {*m*+*n*}convention is useful for looking for incomplete lineages in CVTree branches.
(3) The lineage information of an organism is given in one line with labels *<*D*>*, *<*P*>*, *<*C*>*, *<*O*>*, *<*F*>*, *<*G*>* and *<*S*>*, standing for the ranks domain, phylum, class, order, family, genus and species. The sTrain label *<*T*>* does not appear in lineage information, but may be seen in a leaf. The original lineage information of the built-in genomes was taken from the NCBI taxonomy. The lineage information of user’s genomes was provided at uploading. Users are allowed to make lineage modifications and to see new statistics after doing re-collapsing.
(4) When displaying a tree, the user may pull down a lineage modification window and enter a trial lineage in the form “old_lineage new_lineage”. For example, the initial lineage for *<*T*>*Caldiarchaeum_cryptofilum_OPF8_uid58601 put it in phylum *Thaumarchaeota*, but there is evidence that it belongs to a new phylum, *Aigarchaeota*, so the modification may look like:
  *<*P*>*Thaumarchaeota *· · ·<*G*>*Caldiarchaeum *<*P*>*Aigarchaeota *· · ·<*G*>*Caldiarchaeum
  The modification line is not required to contain all ranks, but the written part must be uniquely recognizable. By submitting the lineage modification, the user performs “re-collapsing” and gets a new report of “convergence statistics”.
(5) The user may select any part of a CVTree and produce a print-quality figure in SVG, EPS, PDF or PNG format.

All of these useful features help to reveal the agreement and discrepancy of a large tree with taxonomy.

## 3. Outline of *Archaea* Taxonomy at and above the Rank Order

The taxonomy of *Archaea* was described in Volume 1 of the Manual, which appeared in 2001 [29], thus being somewhat outdated. Two phyla, the *Crenarchaeota* and the *Euryarchaeota*, were listed there. The *Crenarchaeota* contained only one class, *Thermoprotei*. According to the latest information provided in the *List of Prokaryotic Names with Standing in Nomenclature* (LPSN [30]), the class *Thermoprotei* contains five orders: *Thermoproteales*, *Desulfococcales*, *Sulfolobales*, *Acidilobales* and *Fervidicoccales*, the last two being proposed in 2009 [31] and 2010 [32], respectively. Originally, the phylum, *Euryarchaeota*, contained seven classes: *Methanobacteria*, *Methanococci*, *Halobacteria*, *Thermoplasmata*, *Thermococci*, *Archaeoglobi* and *Methanopyri*; all comprising one order, except for *Methanococci*, which contained three orders. Later on, in a revised roadmap of the Manual [33], the class *Methanococci* was left with only one order; the other two orders became part of the newly proposed class, *Methanomicrobia*. A third order, *Methanocellales*, in the last class was proposed in 2008 [34]. Very recently, there appeared a proposal [35] to divide the single-order class, *Halobacteria*, into three orders. 

Over the past 15 years, a few new archaeal phyla have been proposed: *Korarchaeota* [36,37], *Thaumarchaeota* [38,39,40], *Nanoarchaeota* [41,42,43], *Aigarchaeota* [44], *Parvarchaeota* [45] and *Bathyarchaeota* [46]. All but the last three phyla have been listed in LPSN [30]. We will not touch on *Parvarchaeota* and *Bathyarchaeota*, due to a lack of well-annotated genome data.

The main focus of the present study is to check and compare the positions of these high-rank taxa in CVTree and to compare them with the 16S rRNA sequence analysis where some results obtained by other authors are available.

## 4. Results and Discussion

### 4.1. 16S rRNA Archaeal Phylogeny According to All-Species Living Tree

An authoritative reference to the 16S rRNA phylogeny is the All-Species Living Tree Project (LTP) [47,48,49]. LTP is an ambitious project to construct a single 16S rRNA tree based on all available type strains of hitherto named species of *Archaea* and *Bacteria*. The latest release, LTPs115 [50], of March, 2014, was based on 366 archaeal and 9905 bacterial 16S rRNA sequences. However, the 104-page PDF of the tree is hard to comprehend, especially when it comes to comparing the tree branchings with classification at various taxonomic ranks. We fetched the treeing and lineage information files LPTs115_SSU_tree.newick and LTPs115_SSU.csv from the LTP web site [50] and then collapsed the fully-fledged tree into various taxonomic ranks where possible.

We first obtained the *Archaea* branch containing 366 leaves and collapsed basically to the rank class without doing lineage modification (figure not shown). In fact, it was cut from the original “All-Species Living Tree” LTPs115 [50] based on all 366 archaeal and 9905 bacterial 16S rRNA sequences.

There was a line *<*C*>*Methanomicrobia{71/72} indicating that an outlier violated the monophyly of the branch. By inspecting the figure, the outlier turned out to be:

<O>Unclassified_Methanomicrobia · · · <T>HQ896499 · · · Unclassified_Methanomicrobia

It was located next to the monophyletic *<*C*>*Thermoplasmata{8}. Therefore, it does not look like an “Unclassified_Methanomicrobia”, but might be a miss-classified *Thermoplasmata*. Judging by its close neighborhood, we may temporarily modify the lineage to:

<C>Thermoplasmata<O>Thermoplasmatales<F>Thermoplasmataceae<G>Methanomassiliicoccus· · ·

**Figure 1:**
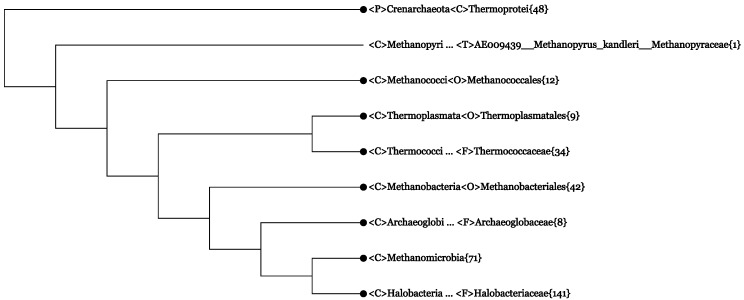
The *Archaea* branch in the All-Species Living Tree based on 366 16S rRNA sequences. The tree has been collapsed to the rank class (*<*C*>*), and only one lineage modification has been made. Numerals in curly brackets indicate the number of sequences contained in a collapsed branch. The collapsing and lineage modification was performed by using a web server similar to CVTree3. This Living Tree Viewer is accessible to all users [51].

After making the lineage modification, we get Figure 1. The branchings in Figure 1 fully agree with the taxonomy of *Archaea*, as outlined in Section 3, at the phylum and class ranks. In particular, the eight classes of *Euryarchaeota* all behave as well-defined monophyletic branches. Further more, if one expands the class *Methanomicrobia*, its three subordinate orders, *Methanocellales*{3}, *Methanosarcinales*{31} and *Methanomicrobiales*{37}, all appear as monophyletic branches (not shown in Figure 1). The definition of orders within *Thermoprotei*, the only class in *Crenarchaeota*, is somehow problematic (more on this point near the end of Subsection 4.2).

This kind of agreement should be expected, as the archaeal taxonomy is largely based on the 16S rRNA sequence analysis. However, as by design, the LTP is restricted to type strains with validly published names, one cannot check the positions of the newly proposed phyla and those strains lacking a definite lineage. The whole-genome-based CVTree approach may complement these aspects of phylogeny, since the criterion for inclusion of a strain into the tree is the availability of a sequenced genome, independent of its standing in nomenclature. In Subsection 4.3, the CVTree results are compared with 16S rRNA analyses done by other authors.

### 4.2. The Whole-Genome-Based CVTree Phylogeny

**Figure 2:**
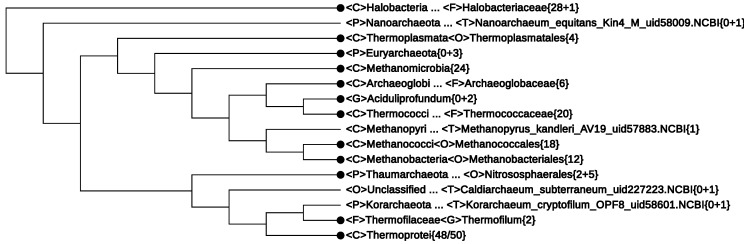
The 179-genome Archaea branch of CVTree obtained by using the CVTree3 web server [21] without making lineage modifications. It has been collapsed to the rank class where possible. The branching order is to be compared with taxonomy, but does not scale the branch lengths.

CVTrees based on 179 *Archaea*, 2850 *Bacteria* and eight *Eukarya* genomes were generated by using the improved version CVTree3 [21] of the web server [20]. We show the *Archaea* part of a big CVTree in Figure 2. When inspecting the figure, we pay more attention to the newly proposed phyla and those taxa with incomplete or suspicious lineage information.

In what follows, the non-monophyletic branches are summarized and possible lineage modifications are suggested.

(1) The first line of Figure 2 *<*F*>*Halobacteriaceae{28+1} informs that among the 29 genomes, there was one without proper lineage information. In fact, it was *Halophilic_archaeon*_DL31_uid72619, a name not validly published and not following the basic rule for a binomen. Its NCBI lineage from phylum down to genus was “unclassified”. However, by expanding this line, the strain is seen to be located deeply inside the class *Halobacteria* (see Figure 4). As at present, the class consists of only one order, which, in turn, is made of one family [33], it is safe to assign this strain to a yet unspecified genus. This modification would yield a monophyletic branch, *Halobacteria*{29}.
(2) The fourth line of Figure 2 *<*P*>*Euryarchaeota{0+3} represents a cluster obtained by collapsing three strains (not explicitly written in the figure):
  - *Thermoplasmatales_archaeon*_BRNA1_uid195930, with NCBI lineage <C>Thermoplasmata<O>Unclassified<F>Unclassified;
  - *Candidatus*_Methanomethylophilus_alvus_Mx1201_uid196597, with NCBI lineage <C>Unclassified<O>Unclassified<F>Unclassified,
  - *Methanomassiliicoccus*_sp_Mx1_Issoire_uid207287, with NCBI lineage <C>Methanomicrobia<O>Unclassified<F>Unclassified.
  If the NCBI lineage would be accepted, two of the above strains must violate the monophyly of the classes *Thermoplasmata*{4/5} and *Methanomicrobia*{24/25}. However, the fact that these three strains, taken together, make a monophyletic branch hint of the possibility to assign them to a yet unspecified class. This modification would restore the monophyly of the two classes *Methanomicrobia*{24} (Line 5 in Figure 2) and *Thermoplasmata*{4} (Line 3 in Figure 2), as seen in Figure 2.
(3) The newly proposed phylum, *Thaumarchaeota*, appears to be non-monophyletic, as an outlying strain, *Candidatus* Caldiarchaeum subterranum, was assigned to this phylum according to the NCBI taxonomy. The NCBI assignment might reflect its position in some phylogenetic tree based on concatenated proteins, e.g., Figure 2 in [52]. However, in the original paper reporting the discovery of this strain [44] and in recent 16S rRNA studies, e.g., [46], *Candidatus* Caldiarchaeum subterranum was proposed to make a new phylum, *Aigarchaeota*. CVTrees support the introduction of this new phylum. A lineage modification of *Candidatus* Caldiarchaeum subterranum from *Thaumarchaeota* to *Aigarchaeota* would lead to a monophyletic *Thaumarchaeota*.
(4) The Candidatus genus, *Aciduliprofundum*, is considered a member of the DHEV2 (deep-sea hydrothermal vent euryarchaeotic 2) phylogenetic cluster. No taxonomic information was given in the original papers [53,54]. The NCBI taxonomy did not provide definite lineage information for this taxon at the class, order and family ranks. According to [53], the whole DHEV2 cluster was located close to *Thermoplasmatales* in a maximum-likelihood analysis of 16S rRNA sequences. A similar placement was seen in [52], where a Bayesian tree of the archaeal domain based on concatenation of 57 ribosomal proteins put a lonely *Aciduliprofundum* next to *Thermoplasmata*. However, in CVTrees, constructed for all *K*-values from three to nine, *Aciduliprofundum* is juxtaposed with the class *Thermococci*{18}. An observation in [54] that this organism shares a rare lipid structure with a few species from *Thermococcales* may hint to its possible association with the latter. If we temporarily presume a lineage:
  <C>Thermococci<O>Unclassified<F>Unclassified<G>Aciduliprofundum · · ·
  one might have a monophyletic class <C>*Thermococci*{20}.
  Since none of the 13 DHEV2 members listed in [53] have a sequenced genome so far, CVTree cannot tell the placement of the DHEV2 cluster as a whole for the time being. It remains an open problem whether DHEV2 is close to *Thermoplasmata* or to *Thermococci* or if a new class is needed to accommodate DHEV2.
(5) The new phylum, *Korarchaeota*, violates the monophyly of the phylum, *Crenarchaeota*, by drawing to itself the family, *Thermofilaceae*. However, in an on-going study of ours (not published yet) using a much larger dataset, this violation no longer shows up; both *Korarchaeota* and *Crenarchaeota* restore their phylum status. Taking into account the fact that both *Korarchaeota* and *Thermofilaceae* are represented by single species for the time being, their placement certainly requires further study with broader sampling of genomes.
  However, it is worth noting that the whole lower cluster of Figure 2 supports a recent proposal for a new “TACK” superphylum [55], made of *Thaumarchaeota*, *Aigarchaeota*, *Crenarchaeota* and *Korarchaeota*.

After making all of the aforementioned lineage modifications, the resulting CVTree (not shown) looks much like Figure 2 with minor changes of some labels.

All eight classes of *Euryarchaeota*, as listed in Section 3, are well-defined on their own. In addition, a new class might be introduced for the three archaeons without detailed lineage information, collapsed as *<*P*>Euryarchaeota*{0+3}. The last point cannot be checked in the All-Species Living Tree without extending it to cover organisms without validly published names.

Now, it comes to inspect the orders in the single-class phylum, *Crenarchaeota*. There is no *a priori* reason to expect that 16S rRNA sequence analysis and the CVTree approach should lead to identical tree branchings. Though all being assigned to *Crenarchaeota*, the forty eight 16S rRNA sequences in the All-Species Living Tree and the 50 genomes in the CVTree do not belong to the same set of organisms. One can only compare those in common.

Two orders, *Sulfolobales* and *Thermoproteales*, are monophyletic in both CVTree and 16S rRNA trees, putting aside the insertion of the single-species, *Korarchaeota*, into *Thermoproteales* in CVTree. The introduction of the new orders, *Acidilobales* in 2009 [31] and *Fervidicoccales* in 2010 [32], violated the monophyly of the so-far monophyletic order, *Desulfurococcales* (the genus, *Acidilobus*, was considered part of *Desulfurococcaceae* before 2009). A main criterion to distinguish species of the new order from that in *Desulfurococcales* was indicated in [31] as acidophily, a point that might require further verification.

The CVTree results summarized above were a continuation and extension of a similar study [56] based on 62 *Archaea* genomes available at the beginning of 2010. The fact that, five years apart and with 117 more genomes added, the results remain consistent informs of the robustness of the CVTree approach.

### 4.3. Phylum Distribution in Other Phylogenies

The conclusions drawn above concerning the positions of the newly proposed phyla and organisms with uncertain lineage information cannot be directly compared with the All-Species Living Tree Project [47,48,49], as by design, LTP only includes strains with validly published names and standing in nomenclature. To this end, one must look for other published studies.

**Figure 3:**
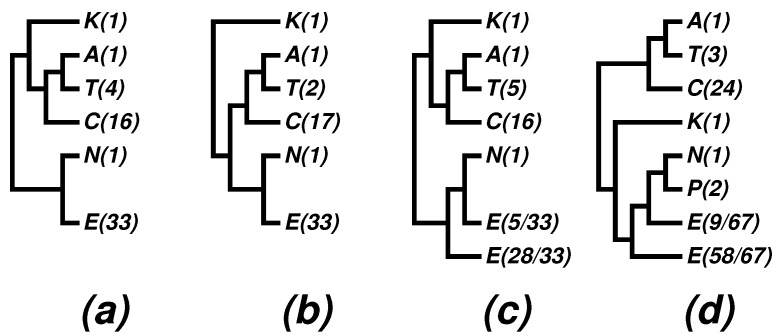
*Archaea* trees collapsed to phyla. Abbreviations: A = *Aigarchaeota*, C = *Crenarchaeota*, E = *Euryarchaeota*, K = *Korarchaeota*, P = *Parvarchaeota*, N = *Nanoarchaeota*, T = *Thaumarchaeota*. (**a–c**) Obtained by collapsing Figure 4 in [44]; (**d**) obtained by collapsing Figure 2 in [52]. Numerals in parentheses indicate the number of species represented in each phylum. For details, see the text and the cited papers.

An effective way of comprehending a tree with many leaves consists of collapsing the tree branches to appropriate taxonomic ranks, as we did in Figure 1 and Figure 2. For published results of other authors, we collapsed their trees manually. Figure 3 shows four such trees collapsed to the phylum level from corresponding trees in [44] and [52]. Figure 3a is a maximum likelihood tree of concatenated SSU and LSU rRNAs using 3063 nucleotide positions; Figure 3b is a maximum likelihood tree of 45 concatenated ribosomal proteins and nine RNA polymerase subunits using 5993 aligned amino acids; and Figure 3c is a maximum likelihood tree from translation EF2 proteins based on 590 residues. All of these three subfigures were obtained by collapsing Figure 4 in [44]. Figure 3d was collapsed from a Bayesian tree based on concatenation of 67 ribosomal proteins from 89 genomes (Figure 2 in [52]).

The interrelationship among phyla deduced from a limited number of representatives in a tree is subject to further changes when more data become available. In 2001, when there was only one genome from each of the bacterial phyla, *Aquificae* and *Thermotogae*, there was speculation that these phyla would make a clade [57,58]. A decade later, it was observed that, though remaining in a big cluster, many other phyla have gotten inserted in between *Aquificae* and *Thermotogae*; see, e.g., [10]. This point concerns especially the archaeal phyla with only one representative genome for the time being.

**Figure 4:**
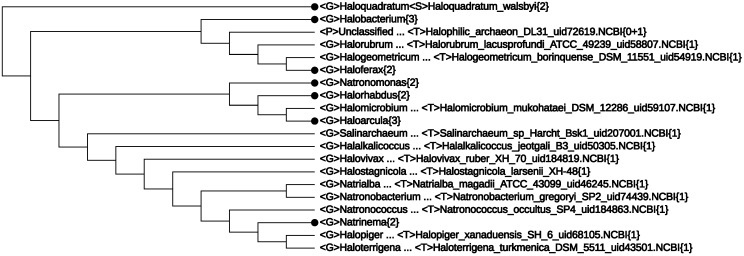
The class, *Halobacteria*, expanded to the genus level.

By comparing our Figure 2 with trees in Figure 3, we see:

(1) The newly proposed phyla, *Thaumarchaeota*, *Korarchaeota* and *Aigarchaeota*, are supported in many phylogenies; especially the superphylum “TACK” is supported in most phylogenies, with “TAC” being a persistent core.
(2) The nano-sized archaean symbiont, *Nanoarchaeum equitans*, has a highly reduced genome (490,885 bp [42]). It is the only described representative of a newly proposed phylum, *Nanoarchaeota*, and it cuts into the otherwise monophyletic phylum, *Euryarchaeota*. We note that the monophyly of *Euryarchaeota* was also violated by *Nanoarchaeum* in some 16S rRNA trees; see, e.g., Figure 4 in a 2009 paper [59], as well as (c) and (d) in Figure 3. It has been known that tiny genomes of endosymbiont microbes often tend to move towards the baseline of a tree and distort the overall picture. In fact, we have suggested skipping such tiny genomes when studying bacterial phylogeny; see, e.g., [28] and a note on the home page of the CVTree web server [20]. In the present case, we may at most say that *Nanoarchaeota* probably makes a separate phylum, but its cutting into *Euryarchaeota* might be a side effect due to the tiny size of the highly-reduced genome.

So far, we have concentrated on “mega-classification” [14] of *Archaea* species, mainly their taxonomy at the rank order and above. Quite recently, there appeared a proposal [35] to split the single-order class, *Halobacteria*, into three orders: *Haloferacales*, *Natrialbales* and *Halobacteriales*. In order to check whether CVTree supports this proposal or not, an expansion of the class, *Halobacteria*{29}, the first line in Figure 2, is given in Figure 4. Indeed, the three main branches are clearly seen in Figure 4, corresponding to the three proposed orders, except for a single genus, *Halakalicoccus*, which did not take a definite position, even in trees obtained by different methods in [35]. Being supported by the previous predictive power of CVTree, we anticipate that the position of *Halakalicoccus* in Figure 4 may better reflect the reality, a point verifiable in the future.

## 5. Conclusions

The CVTree approach to prokaryotic phylogeny distinguishes itself from the 16S rRNA sequence analysis, both in the input data (genomes instead of RNA sequences) and in the methodology (*K*-peptide counting *versus* sequence alignment). The agreement of the two approaches makes the results more objective and convincing, whereas a few discrepancies call for further study. A phylogenetic study across many phyla naturally places emphasis on building a robust backbone for classification. At taxonomic rank order and above, whole-genome approaches are essentially simpler, as the only prerequisite is having the genomes at hand. Sooner or later, phylogenetic information and taxonomic placement will become by-products of genome analyses. The cost of sequencing a prokaryotic genome will drop below the average expense of carrying out conventional phenotyping experiments. To this end, a crucial factor is the availability of reliable, convenient and easy-to-use tools, such as the CVTree web server. The technique of collapsing and expanding tree branches with an interactive display, as well as automatic reporting of comparison results at all taxonomic ranks makes large-scale studies more feasible. The experience accumulated in this study on 179 archaeal strains will be instructive for carrying out similar studies on *Bacteria*, which would cover hundred-fold more strains.

The 16S rRNA sequence analysis will remain an indispensable tool in microbiology. The number of sequenced genomes can never catch up with that of rRNA sequences. Although the CVTree method adds more agreement than discrepancy to the 16S rRNA results, the difference between the two approaches certainly deserves in-depth scrutiny. In addition, since high resolution power at the species level and below is a prominent advantage of CVTree as compared to 16S rRNA sequence analysis [12,60], we will elaborate on this aspect in the future when the amount of sequenced archaeal genomes will have increased substantially.

## Appendix: List of Genomes Used in This Study

All of the 179 genomes used in the present study are listed in the following table, together with their accession number and approximate proteome size (in 106 amino acids). The 165 genomes from the NCBI FTP site [19] come with uid numbers, but the uploaded ones appear without uid. We note that in the EBI list of *Archaea* [22], there are 176 species. Excluding a tiny one, 175 genomes remain. The four genomes present at NCBI, but absent at EBI, are Nos. 31*,* 40*,* 106 and 137.

**Table A1:** list of genomes used in this study.

No.	Name of Strain	Proteome Size (10^6^AA)	Accession Number
1	Acidianus hospitalis W1 uid66875	0.62	NC_015518
2	Acidilobus saccharovorans 345 15 uid51395	0.45	NC_014374
3	Aciduliprofundum boonei T469 uid43333	0.47	NC_013926
4	Aciduliprofundum sp. MAR08 339 uid184407	0.45	NC_019942
5	Aeropyrum camini SY1 JCM 12091 uid222311	0.47	NC_022521
6	Aeropyrum pernix K1 uid57757	0.49	NC_000854
7	Archaeoglobus fulgidus DSM 4304 uid57717	0.67	NC_000917
8	Archaeoglobus fulgidus DSM 8774	0.69	CP006577
9	Archaeoglobus profundus DSM 5631 uid43493	0.48	NC_013741
10	Archaeoglobus sulfaticallidus PM70 1 uid201033	0.61	NC_021169
11	Archaeoglobus veneficus SNP6 uid65269	0.56	NC_015320
12	Caldisphaera lagunensis DSM 15908 uid183486	0.44	NC_019791
13	Caldivirga maquilingensis IC 167 uid58711	0.60	NC_009954
14	Candidatus Caldiarchaeum subterraneum uid227223	0.51	NC_022786
15	Candidatus Korarchaeum cryptofilum OPF8 uid58601	0.48	NC_010482
16	Candidatus Methanomethylophilus alvus Mx1201 uid196597	0.49	NC_020913
17	Candidatus Nitrosopumilus koreensis AR1 uid176129	0.47	NC_018655
18	Candidatus Nitrosopumilus sp. AR2 uid176130	0.49	NC_018656
19	Candidatus Nitrososphaera evergladensis SR1	0.82	CP007174
20	Candidatus Nitrososphaera gargensis Ga9 2 uid176707	0.77	NC_018719
21	Methanomassiliicoccus sp. Mx1 Issoire uid207287	0.56	NC_021353
22	Cenarchaeum symbiosum A uid61411	0.62	NC_014820
23	Desulfurococcus fermentans DSM 16532 uid75119	0.40	NC_018001
24	Desulfurococcus kamchatkensis 1221n uid59133	0.40	NC_011766
25	Desulfurococcus mucosus DSM 2162 uid62227	0.39	NC_014961
26	Ferroglobus placidus DSM 10642 uid40863	0.66	NC_013849
27	Ferroplasma acidarmanus fer1 uid54095	0.57	NC_021592
28	Fervidicoccus fontis Kam940 uid162201	0.38	NC_017461
29	Halalkalicoccus jeotgali B3 uid50305	0.83	NC_014297
30	Haloarcula hispanica ATCC 33960 uid72475	1.00	NC_0159432
31	Haloarcula hispanica N601 uid230920	0.98	NC_0230102
32	Haloarcula marismortui ATCC 43049 uid57719	0.97	NC_0063972
33	Halobacterium salinarum R1 uid61571	0.60	NC_010364
34	Halobacterium sp. DL1	0.83	CP007060
35	Halobacterium sp. NRC 1 uid57769	0.59	NC_002607
36	Haloferax mediterranei ATCC 33500 uid167315	0.84	NC_017941
37	Haloferax volcanii DS2 uid46845	0.82	NC_013967
38	Halogeometricum borinquense DSM 11551 uid54919	0.82	NC_014729
39	Halomicrobium mukohataei DSM 12286 uid59107	0.90	NC_013202
40	Halophilic archaeon DL31 uid72619	0.81	NC_015954
41	Halopiger xanaduensis SH 6 uid68105	1.05	NC_015666
42	Haloquadratum walsbyi C23 uid162019	0.77	NC_017459
43	Haloquadratum walsbyi DSM 16790 uid58673	0.78	NC_008212
44	Halorhabdus tiamatea SARL4B uid214082	0.79	NC_021921
45	Halorhabdus utahensis DSM 12940 uid59189	0.91	NC_013158
46	Halorubrum lacusprofundi ATCC 49239 uid58807	0.93	NC_0120292
47	Halostagnicola larsenii XH-48	0.78	CP007055
48	Haloterrigena turkmenica DSM 5511 uid43501	1.09	NC_013743
49	Halovivax ruber XH 70 uid184819	0.91	NC_019964
50	Hyperthermus butylicus DSM 5456 uid57755	0.45	NC_008818
51	Ignicoccus hospitalis KIN4 I uid58365	0.40	NC_009776
52	Ignisphaera aggregans DSM 17230 uid51875	0.54	NC_014471
53	Metallosphaera cuprina Ar 4 uid66329	0.54	NC_015435
54	Metallosphaera sedula DSM 5348 uid58717	0.64	NC_009440
55	Methanobacterium formicicum strain BRM9	0.67	CP006933
56	Methanobacterium sp. AL 21 uid63623	0.72	NC_015216
57	Methanobacterium sp. MB1 complete sequence uid231690	0.56	NC_023044
58	Methanobacterium sp. SWAN 1 uid67359	0.66	NC_015574
59	Methanobrevibacter ruminantium M1 uid45857	0.76	NC_013790
60	Methanobrevibacter smithii ATCC 35061 uid58827	0.56	NC_009515
61	Methanobrevibacter sp. AbM4 uid206516	0.50	NC_021355
62	Methanocaldococcus fervens AG86 uid59347	0.44	NC_013156
63	Methanocaldococcus infernus ME uid48803	0.41	NC_014122
64	Methanocaldococcus jannaschii DSM 2661 uid57713	0.48	NC_000909
65	Methanocaldococcus sp. JH146	0.47	CP009149
66	Methanocaldococcus sp. FS406 22 uid42499	0.51	NC_013887
67	Methanocaldococcus vulcanius M7 uid41131	0.49	NC_013407
68	Methanocella arvoryzae MRE50 uid61623	0.89	NC_009464
69	Methanocella conradii HZ254 uid157911	0.70	NC_017034
70	Methanocella paludicola SANAE uid42887	0.86	NC_013665
71	Methanococcoides burtonii DSM 6242 uid58023	0.69	NC_007955
72	Methanococcus aeolicus Nankai 3 uid58823	0.44	NC_009635
73	Methanococcus maripaludis C5 uid58741	0.51	NC_009135
74	Methanococcus maripaludis C6 uid58947	0.51	NC_009975
75	Methanococcus maripaludis C7 uid58847	0.51	NC_009637
76	Methanococcus maripaludis KA1 DNA	0.55	AP011526
77	Methanococcus maripaludis OS7 DNA	0.52	AP011528
78	Methanococcus maripaludis S2 uid58035	0.49	NC_005791
79	Methanococcus maripaludis X1 uid70729	0.51	NC_015847
80	Methanococcus vannielii SB uid58767	0.49	NC_009634
81	Methanococcus voltae A3 uid49529	0.51	NC_014222
82	Methanocorpusculum labreanum Z uid58785	0.52	NC_008942
83	Methanoculleus bourgensis MS2T uid171377	0.77	NC_018227
84	Methanoculleus marisnigri JR1 uid58561	0.72	NC_009051
85	Methanohalobium evestigatum Z 7303 uid49857	0.63	NC_014253
86	Methanohalophilus mahii DSM 5219 uid47313	0.59	NC_014002
87	Methanolobus psychrophilus R15 uid177925	0.87	NC_018876
88	Methanomethylovorans hollandica DSM 15978 uid184864	0.69	NC_019977
89	Methanoplanus petrolearius DSM 11571 uid52695	0.83	NC_014507
90	Methanopyrus kandleri AV19 uid57883	0.50	NC_003551
91	Methanoregula boonei 6A8 uid58815	0.73	NC_009712
92	Methanoregula formicicum SMSP uid184406	0.81	NC_019943
93	Methanosaeta concilii GP6 uid66207	0.84	NC_015416
94	Methanosaeta harundinacea 6Ac uid81199	0.73	NC_017527
95	Methanosaeta thermophila PT uid58469	0.51	NC_008553
96	Methanosalsum zhilinae DSM 4017 uid68249	0.61	NC_015676
97	Methanosarcina acetivorans C2A uid57879	1.42	NC_003552
98	Methanosarcina barkeri str Fusaro uid57715	1.12	NC_007355
99	Methanosarcina mazei Go1 uid57893	1.02	NC_003901
100	Methanosarcina mazei Tuc01 uid190185	0.82	NC_020389
101	Methanosphaera stadtmanae DSM 3091 uid58407	0.49	NC_007681
102	Methanosphaerula palustris E1 9c uid59193	0.82	NC_011832
103	Methanospirillum hungatei JF 1 uid58181	1.01	NC_007796
104	Methanothermobacter marburgensis str Marburg uid51637	0.49	NC_014408
105	Methanothermobacter thermautotrophicus str Delta H uid57877	0.53	NC_000916
106	Methanothermobacter thermautotrophicus CaT2 DNA	0.51	AP011952
107	Methanothermococcus okinawensis IH1 uid51535	0.45	NC_015636
108	Methanothermus fervidus DSM 2088 uid60167	0.38	NC_014658
109	Methanotorris igneus Kol 5 uid67321	0.51	NC_015562
110	Nanoarchaeum equitans Kin4 M uid58009	0.15	NC_005213
111	Natrialba magadii ATCC 43099 uid46245	1.05	NC_013922
112	Natrinema pellirubrum DSM 15624 uid74437	1.06	NC_019962
113	Natrinema sp. J7 2 uid171337	1.05	NC_018224
114	Natronobacterium gregoryi SP2 uid74439	1.04	NC_019792
115	Natronococcus occultus SP4 uid184863	1.12	NC_019974
116	Natronomonas moolapensis 8 8 11 uid190182	0.82	NC_020388
117	Natronomonas pharaonis DSM 2160 uid58435	0.78	NC_007426
118	Nitrosopumilus maritimus SCM1 uid58903	0.49	NC_010085
119	Nitrososphaera viennensis EN76	0.73	CP007536
120	Palaeococcus pacificus DY20341	0.56	CP006019
121	Picrophilus torridus DSM 9790 uid58041	0.47	NC_005877
122	Pyrobaculum aerophilum str IM2 uid57727	0.66	NC_003364
123	Pyrobaculum arsenaticum DSM 13514 uid58409	0.61	NC_009376
124	Pyrobaculum calidifontis JCM 11548 uid58787	0.61	NC_009073
125	Pyrobaculum islandicum DSM 4184 uid58635	0.53	NC_008701
126	Pyrobaculum neutrophilum V24Sta uid58421	0.53	NC_010525
127	Pyrobaculum oguniense TE7 uid84411	0.71	NC_016885
128	Pyrobaculum sp. 1860 uid82379	0.73	NC_016645
129	Pyrococcus abyssi GE5 uid62903	0.54	NC_000868
130	Pyrococcus furiosus COM1 uid169620	0.57	NC_018092
131	Pyrococcus furiosus DSM 3638 uid57873	0.59	NC_003413
132	Pyrococcus horikoshii OT3 uid57753	0.55	NC_000961
133	Pyrococcus sp. NA2 uid66551	0.57	NC_015474
134	Pyrococcus sp. ST04 uid167261	0.52	NC_017946
135	Pyrococcus yayanosii CH1 uid68281	0.51	NC_015680
136	Pyrolobus fumarii 1A uid73415	0.54	NC_015931
137	Salinarchaeum sp. Harcht Bsk1 uid207001	0.91	NC_021313
138	Staphylothermus hellenicus DSM 12710 uid45893	0.46	NC_014205
139	Staphylothermus marinus F1 uid58719	0.46	NC_009033
140	Sulfolobus acidocaldarius DSM 639 uid58379	0.63	NC_007181
141	Sulfolobus acidocaldarius N8 uid189027	0.62	NC_020246
142	Sulfolobus acidocaldarius Ron12 I uid189028	0.64	NC_020247
143	Sulfolobus acidocaldarius SUSAZ uid232254	0.59	NC_023069
144	Sulfolobus islandicus HVE10 4 uid162067	0.76	NC_017275
145	Sulfolobus islandicus L D 8 5 uid43679	0.77	NC_013769
146	Sulfolobus islandicus L S 2 15 uid58871	0.76	NC_012589
147	Sulfolobus islandicus LAL14 1 uid197216	0.71	NC_021058
148	Sulfolobus islandicus M 14 25 uid58849	0.74	NC_012588
149	Sulfolobus islandicus M 16 27 uid58851	0.76	NC_012632
150	Sulfolobus islandicus M 16 4 uid58841	0.75	NC_012726
151	Sulfolobus islandicus REY15A uid162071	0.72	NC_017276
152	Sulfolobus islandicus Y G 57 14 uid58923	0.78	NC_012622
153	Sulfolobus islandicus Y N 15 51 uid58825	0.77	NC_012623
154	Sulfolobus solfataricus 98 2 uid167998	0.72	NC_017274
155	Sulfolobus solfataricus P2 uid57721	0.84	NC_002754
156	Sulfolobus tokodaii str 7 uid57807	0.76	NC_003106
157	Thermococcus barophilus MP uid54733	0.62	NC_014804
158	Thermococcus eurythermalis strain A501	0.60	CP008887
159	Thermococcus gammatolerans EJ3 uid59389	0.64	NC_012804
160	Thermococcus kodakarensis KOD1 uid58225	0.64	NC_006624
161	Thermococcus litoralis DSM 5473 uid82997	0.67	NC_022084
162	Thermococcus nautili strain 30 1	0.61	CP007264
163	Thermococcus onnurineus NA1 uid59043	0.56	NC_011529
164	Thermococcus sibiricus MM 739 uid59399	0.55	NC_012883
165	Thermococcus sp. 4557 uid70841	0.61	NC_015865
166	Thermococcus sp. AM4 uid54735	0.63	NC_016051
167	Thermococcus sp. CL1 uid168259	0.58	NC_018015
168	Thermococcus sp. ES1	0.58	CP006965
169	Thermofilum pendens Hrk 5 uid58563	0.54	NC_008698
170	Thermofilum sp. 1910b uid215374	0.52	NC_022093
171	Thermogladius cellulolyticus 1633 uid167488	0.41	NC_017954
172	Thermoplasma acidophilum DSM 1728 uid61573	0.45	NC_002578
173	Thermoplasma volcanium GSS1 uid57751	0.45	NC_002689
174	Thermoplasmatales archaeon BRNA1 uid195930	0.44	NC_020892
175	Thermoproteus tenax Kra 1 uid74443	0.55	NC_016070
176	Thermoproteus uzoniensis 768 20 uid65089	0.59	NC_015315
177	Thermosphaera aggregans DSM 11486 uid48993	0.40	NC_014160
178	Vulcanisaeta distributa DSM 14429 uid52827	0.71	NC_014537
179	Vulcanisaeta moutnovskia 768 28 uid63631	0.67	NC_015151
